# Role of ECMO and percutaneous mechanical thrombectomy in the management of acute pulmonary embolism

**DOI:** 10.3389/fcvm.2026.1935374

**Published:** 2026-08-20

**Authors:** Mark G. Davies, Joseph P. Hart

**Affiliations:** 1 Center for Quality, Effectiveness, and Outcomes in Cardiovascular Diseases, Houston, TX, United States; 2 Department of Vascular and Endovascular Surgery, Ascension Health, Waco, TX, United States; 3 Division of Vascular Surgery, Medical College of Wisconsin, Milwaukee, WI, United States; 4 Clement J. Zablocki Medical Veterans Administration Medical Center, Milwaukee, WI, United States

**Keywords:** ECMO, outcomes, percutaneous mechanical therapy, pulmonary embolism, intervention

## Abstract

Interventional treatment for hemodynamically unstable pulmonary embolism (PE) is now common due to the fact that unstable PE carries a significant 30-day mortality. Unstable PE is characterized by acute right ventricular failure, hypotension, and hypoxia, leading to cardiovascular collapse and cardiac arrest. Implementation of veno-arterial extracorporeal membrane oxygenation (VA-ECMO) acutely provides oxygenation support to improve hypoxia and offload the right ventricular (RV) pressure in the belief that rapid reduction of hypoxia and right ventricular (RV) pressure will provide a window for intervention. There are two strategies for using VA-ECMO during percutaneous pulmonary mechanical thrombectomy: VA-ECMO-assisted mechanical thrombectomy and mechanical thrombectomy with standby VA-ECMO. While the application of VA-ECMO in percutaneous mechanical thrombectomy is usually short-term, providers must be cognizant that it can be associated with substantial multisystem morbidity due to a systemic inflammatory response, hemorrhagic stroke, renal dysfunction, and bleeding, and these factors will impact outcomes. The evolving paradigm positions VA-ECMO combined with percutaneous pulmonary mechanical thrombectomy as the preferred combination for the high-risk PE patients (acute right ventricular failure, refractory shock, and cardiac arrest), while percutaneous pulmonary mechanical thrombectomy alone with standby VA-ECMO suffices for most intermediate PE patients.

## Introduction

Acute pulmonary embolism (PE) continues to be a leading cause of preventable cardiopulmonary mortality and morbidity (1). This current spectrum of acute PE has been refined by the European Society of Cardiology (ESC and focuses on the mortality risk associated with an acute PE event: “low”, “intermediate,” and “high” (2). High-risk acute pulmonary embolism accounts for approximately 5% of patients diagnosed with pulmonary embolism (3). While hemodynamically stable patients with a PE have a 30-day mortality of 1%–2% if they remain hemodynamically stable, but the 30-day mortality increases to 10% to 25% for hemodynamically unstable patients, and in those patients who have a cardiac arrest their 30-day mortality exceeds 50% (4–8). Deployment of Veno-Arterial Extra-Corporal Membrane Oxygenation (VA-ECMO) in high-risk, hemodynamically unstable patients provides oxygenation and hemodynamic support to address hypoxia and an offloading strategy for right heart circulation, both contributing to the shock that develops in high-risk PE (9, 10). Once a patient is hemodynamically stabilized, a percutaneous thrombectomy can be accomplished. This narrative review examines the current state of integrating VA-ECMO with percutaneous pulmonary thrombectomy in critically ill patients with acute PE.

### Acute pulmonary embolism

The presence of an acute PE leads to significant alterations in hemodynamic parameters within the pulmonary circulation, compromises pulmonary gas exchange, and alters pulmonary mechanics (9, 11–13). As a result, more than half of patients present with hypoxemia (PaO_2_ < 70 mm Hg) (11, 14). While hypoxia is common and treatable, most early deaths are attributed to acute right ventricular (RV) pressure overload and subsequent ventricular failure as a result of a rapid uncompensated increase in pulmonary vascular resistance (15). The increase in pulmonary vascular resistance seen in PE results from direct obstruction of the pulmonary arterial branches, which is further exacerbated by hypoxia- and acidosis-induced vasoconstriction due to the release of vasoactive mediators from the pulmonary vessel wall. High-risk acute PE occurs when large-volume emboli obstruct significant portions of the pulmonary arterial tree, leading to sudden hemodynamic instability. Hemodynamic instability is now defined as systolic blood pressure < 90 mmHg; hypotension requiring vasopressor support; a decrease in systolic blood pressure > 40 mmHg for ≥15 min requiring inotropic support; pulselessness; or persistent profound bradycardia (16–18).

### Classification

The American Heart Association (AHA) introduced the original concept of three distinct acute pulmonary embolism events based on their respective anatomic and physiological findings: “Minor” to “Sub-massive” to “Massive” PE (16). This classification system was advanced and modified by a classification system adopted by the European Society of Cardiology (ESC), which focused on the associated risk of mortality from any acute pulmonary embolism event: “high,” “intermediate,” and “low” (2) (Table 1). In the High-risk acute pulmonary embolism category, hemodynamic instability was defined as a systolic blood pressure < 90 mmHg, hypotension requiring vasopressor support, or a decrement of the systolic blood pressure > 40 mmHg for ≥15 min or requiring inotropic support, pulselessness, or persistent profound bradycardia (16–18).. In 2026, a new AHA joint societies guideline was published that introduced a new clinical classification scheme for acute pulmonary embolism, titled “Acute Pulmonary Embolism Clinical Categories,” which offered 5 categories (A-E) and subcategories, ranging from low to high risk for adverse outcomes (Table 1) (19). Currently, there are no reports using the new AHA joint societies guideline.

**Table 1:** ESC and AHA classification of acute pulmonary embolism (PE).

ESC Clinical Categories	
Category	Description
Low-risk	Patients do not require oxygen, show no signs of right ventricular (RV) dysfunction, and have normal biomarkers.
Intermediate-low risk	Patients have either elevated biomarkers or RV dysfunction, but not both.
Intermediate-high risk	Patients exhibit both elevated biomarkers and RV dysfunction.
High-risk	Patients experience prolonged hypotension (systolic BP <90 mmHg for at least 15 min), require pressor support, or are in cardiac arrest.

AHA Clinical Categories		
Category	Description	Risk Level
A	Subclinical PE; incidental finding on imaging	Low risk
B	Symptomatic but low clinical severity score (PESI class I-II, sPESI=0, Hestia=0)	Low risk
C	Symptomatic with elevated severity score (PESI class III-V, sPESI ≥1, Hestia ≥1)	Moderate risk
D	Incipient cardiopulmonary failure (normotensive shock)	High risk
E	Cardiopulmonary failure	Very high risk

ESC, European Society for Cardiology; AHA, American Heart Association; PESI, Pulmonary Embolism Severity Index; sPESI, simplified Pulmonary Embolism Severity Index.

### Current guidelines and VA-ECMO

The American College of Chest Physicians (ACCP), the European Society of Cardiology (ESC), and the American Heart Association have developed and published evidence-based guidance and recommendations for therapy across the spectrum of acute PE (2, 19, 20). In hemodynamically compromised individuals, recommended therapeutic approaches include systemic thrombolysis. catheter-based techniques (catheter-directed thrombolysis (CDT) and mechanical thrombectomy (MT)) and ultimately surgical embolectomy. The 2019 American Heart Association and 2019 European Society of Cardiology guidelines on high-risk PE have proposed that patients requiring direct therapeutic escalation (systemic thrombolysis, catheter-directed thrombolysis, or surgical embolectomy) should be considered as candidates for VA-ECMO in conjunction with one or more of these interventions to support patients suffering refractory cardiogenic shock or cardiac arrest. The 2026 American Heart Association recommends VA-ECMO for the patient in cardiopulmonary failure (category E) (19),

### Percutaneous pulmonary thrombectomy

Mechanical circulatory support may be of benefit for patients in cardiogenic shock as a consequence of known or suspected PE with evidence of RV dysfunction. Use of these supportive measures will depend on local resources and expertise, but most commonly includes VA-ECMO. VA-ECMO serves to rapidly decrease RV preload while delivering oxygenated blood to peripheral tissues and end organs, thus halting the deleterious effects of hypoxia and cardiogenic shock. Risks and benefits of continued anticoagulation while on mechanical circulatory support should be constantly re-evaluated. Data are limited on the benefit of advanced catheter-based therapies (for patients with acute PE who are being supported with VA-ECMO.

Mechanical thrombectomy appears to offer superior performance to anticoagulation alone in selected patients with acute PE. In the most recent systematic review and meta-analysis by Chan et al. (21), the authors identified seven studies of patients with intermediate-risk PE in whom pulmonary mechanical thrombectomy (MT) was compared to Anticoagulation alone. MT was associated with a significantly lower rate of all-cause 30-day mortality (21). There was no difference in all-cause in-hospital mortality, hospital length of stay or ICU length of stay. This study corroborates similar findings on outcomes in several meta-analyses published in prior years that compared mechanical thrombectomy and anticoagulation in acute PE (22–24).

In an analysis of 10 studies, Singh et al. (25) demonstrated that compared to the CDT group, patients undergoing MT had longer procedure time, longer fluoroscopy duration, and greater estimated blood loss, but with a lower postprocedural ICU admission rate and shorter ICU length of stay. However, there were no significant differences found between the two modalities with respect to changes in mean pulmonary arterial pressure and RV/LV ratio, hospital length of stay, intracranial hemorrhage, major bleeding, all-cause mortality rates, PE-related readmission rates, and all-cause readmission rates. Ismayl et al. reported similar outcomes (26)

Chandra et al. conducted a systematic review and meta-analysis of MT in acute PE (27). Fourteen case series were identified, comprising 516 patients with a mean age of 58 years. However, in this dataset, only three studies had patients with high-risk PE, two studies had only intermediate-risk PE, and the remaining nine studies had a combination of both high-risk and intermediate-risk PE. The meta-analyses showed that in-hospital mortality was 3.6% (0.7%, 7.9%,95% CI), major bleeding occurred in 0.5% (0.0%, 1.8%], technical success was achieved in 97.1% (94.8%, 98.4%), and clinical success (if the therapeutic goal was achieved) was reported in 90.7% (85.5%, 94.3%) of patients.

### VA-ECMO

Extra-Corporeal Membrane Oxygenation (ECMO) is a form of partial cardiopulmonary bypass, which permits short-term support of respiratory and cardiac function in critically ill patients who are experiencing a reversible cardiopulmonary crisis. In the environment of acute pulmonary embolism care, ECMO is deployed as a veno-arterial circuit, which allows for deoxygenated blood to be removed from a central venous or a right atrial catheter, passed through a bedside oxygenator, and then returned fully oxygenated into the body by way of a central or a peripheral artery catheter. The description of the VA-ECMO circuit and the complications associated with VA-ECMO have been described previously (28–30)**.** The reported outcomes for EMCO therapy for PE are limited by study heterogeneity, multiple confounding and selection biases, and limited generalizability. In 2015, Yusuff et al. (31) reported that patients presenting with hemodynamically unstable PE had an overall survival of 70.1%. Survival on VA-ECMO was not influenced by the advanced intervention used to treat the PE. Importantly, the authors noted that patients who suffered cardiac arrest had much lower survival rates than those who did not. Baldetti and colleagues (2020 showed that, in patients placed on VA-ECMO for hemodynamically unstable PE, the early all-cause mortality was 41%, but only 57% of patients underwent adjunctive reperfusion therapies. In 2021, Harwood Scott et al. (32) showed that only sixty-one percent of patients presenting with cardiac arrest due to PE will survive to discharge. In the same year, Kaso et al. (33) showed that In-hospital mortality did not differ significantly between patients with hemodynamically unstable PE and/or cardiac arrest treated with or without VA-ECMO. In an updated analysis from 2024, Boey et al. examined patients treated with VA-ECMO and catheter-directed therapy (29%%) and demonstrated significantly lower mortality compared with those treated with ECMO and systemic thrombolysis alone (57%). Importantly, these authors noted that the pooled duration of VA-ECMO was only 4.4 days. Based on these varied meta-analyses, it appears that initiating VA-ECMO alone, with or without systemic thrombolysis, does not improve outcomes compared with conventional therapy, and all authors emphasize that VA-ECMO should be followed by mechanical thrombus removal if and when possible.

### Combining ECMO and percutaneous therapy

**Figure 1:**
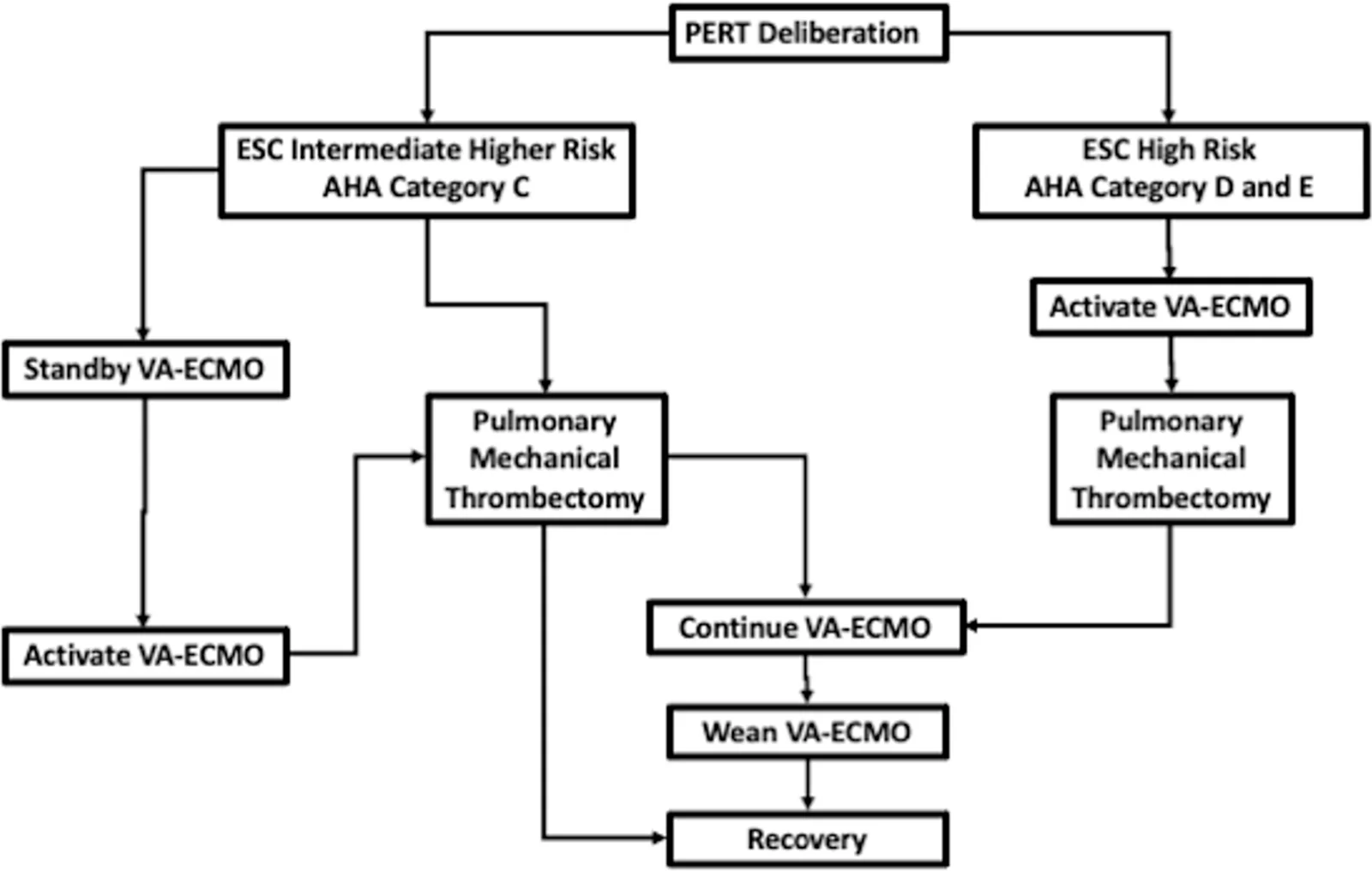
Deployment paradigms of VA–ECMO and mechanical thrombectomy following a PERT consultation, the patient is evaluated and assigned to a risk category. For pulmonary embolism in the ESC intermediate-high risk category (AHA category C) or the ESC high-risk category (AHA categories D and E), VA-ECMO is initiated, and a MT is performed. In the potentially unstable patient, VA-ECMO is kept on standby, and an MT is performed. If the patient becomes unstable, VA-ECMO is initiated. In both scenarios, VA-ECMO is continued until the patient is stable and can be weaned from hemodynamic support. AHA, American Heart Association; ESC, European Society of Cardiology; MT, Mechanical thrombectomy; PERT, Pulmonary Embolism Response Team; VA-ECMO, Veno-Arterial Extracorporeal Membrane Oxygenation.

The primary indication for ECMO is the short-term treatment of PE-induced cardiogenic shock and witnessed cardiac arrest. The secondary indication for ECMO is to treat hemodynamic compromise during the mechanical thrombectomy procedure. The goal of VA-ECMO in PE management is to stabilize the patient by restoring circulation, offloading the right ventricle, and restoring end-organ oxygenation, or to restore circulation during or after an intervention for PE. Once hemodynamic stability is present, the goal of percutaneous thrombectomy therapy is to remove sufficient clot to restore hemodynamically stable pulmonary circulation and RV function (Table 2). There are two integrated approaches that combine mechanical thrombectomy and ECMO, and are dependent on patient stability (Figure 1):

- *ECMO-Supported Pulmonary Mechanical Thrombectomy***:** This approach is used when the patient is in refractory shock or has been resuscitated after a cardiac arrest. VA-ECMO is initiated immediately via femoral cannulation. Once systemic perfusion is guaranteed, a large-bore aspiration mechanical thrombectomy catheter is introduced into the pulmonary arteries, often through the contralateral vein from the venous access used for VA-ECMO circuit.
- *Pulmonary Mechanical thrombectomy with VA-ECMO on Standby***:** For currently hemodynamically stable or stabilized patients with intermediate higher risk and high-risk PE, clinicians obtain arterial and venous femoral access sites and have an ECMO circuit primed and ready. If the patient codes or decompensates during the thrombectomy, VA-ECMO is deployed within minutes, and the case switches to VA-ECMO-supported Pulmonary Mechanical Thrombectomy.

**Table 2:** Benefits of VA-ECMO and mechanical thrombectomy in hemodynamically unstable pulmonary embolism.

VA-ECMO	Mechanical thrombectomy
Restores systemic perfusion	Removes obstructing clot
Supports oxygenation	Reduces RV afterload
Supports RV failure	Improves pulmonary artery flow
Allows stabilization before intervention	Avoids systemic thrombolysis
Bridge to recovery or surgery	Immediate reperfusion

Simultaneous VA-ECMO support and percutaneous mechanical thrombectomy (MT) require careful planning for venous access to minimize catheter interactions while maintaining adequate ECMO flow. In most cases, the femoral vein used for venous drainage during VA-ECMO should be kept separate from the thrombectomy access site, making contralateral femoral venous access the preferred strategy whenever anatomy permits. This approach reduces competition for vessel diameter, facilitates independent catheter manipulation, and decreases the risk of thrombectomy devices interfering with the ECMO drainage cannula. Ipsilateral femoral venous access may be necessary in patients with limited venous access or prior instrumentation, but it requires careful positioning of large-bore sheaths under fluoroscopic guidance to avoid mechanical interaction between the catheters, venous obstruction, or difficulty advancing aspiration catheters. The simultaneous presence of multiple large-bore venous sheaths (typically 22–29 Fr ECMO drainage cannulas and 20–26 Fr thrombectomy sheaths) within the IVC may reduce venous return, increase access-site bleeding, predispose to iliofemoral venous injury or thrombosis, and transiently impair ECMO drainage due to catheter competition: the “suck-down” phenomenon (34). The “suck-down” phenomenon (also called chattering or venous collapse) is a well-recognized complication during VA- or VV-ECMO in which the negative pressure generated by the centrifugal pump causes the wall of the vena cava or right atrium to collapse against the drainage cannula. This transient obstruction reduces venous return into the ECMO circuit, resulting in abrupt decreases in pump flow and hemodynamic instability. The current paradigm for treatment of this complication focuses on restoring venous return. ECMO pump speed can be reduced temporarily while intravenous fluids are administered if preload is inadequate. If the “suck-down” phenomenon persists, the drainage cannula can be repositioned under fluoroscopic or echocardiographic guidance, or the thrombectomy sheath or aspiration catheter can be withdrawn or repositioned if mechanical interference is suspected. To avoid these issues during mechanical thrombectomy, continuous monitoring of ECMO flow, circuit pressures, and systemic hemodynamics during thrombectomy is essential, with repositioning of cannulas or temporary adjustment of pump flow occasionally required to maintain adequate circulatory support while allowing effective clot extraction. Although published experience remains limited, these technical considerations have been consistently described in contemporary case series and expert recommendations involving combined VA-ECMO and catheter-based therapy for high-risk pulmonary embolism*.*

Following successful mechanical thrombectomy, VA-ECMO should not be discontinued or weaned solely on the basis of angiographic improvement. Rather, weaning should occur only after the patient has demonstrated sustained recovery of right ventricular (RV) function and cardiopulmonary stability. Key parameters supporting ECMO weaning include normalization or substantial improvement of the RV/LV diameter ratio (typically <1.0), recovery of RV systolic performance as evidenced by improvement in tricuspid annular plane systolic excursion (TAPSE) (generally ≥16–17 mm), reduced RV dilation and septal flattening, and improved RV fractional area change or tissue Doppler S' velocity (35–39). Additional hemodynamic indicators that support weaning include reduced mean pulmonary artery pressure, stabilization of systemic arterial pressure with minimal or no vasopressor support, normalization of cardiac index and mixed venous oxygen saturation, and reduced lactate production, suggesting recovered tissue perfusion. Successful weaning also requires adequate gas exchange with acceptable ventilator settings, stable ECMO flow during trial weans, and no recurrence of RV failure or hemodynamic deterioration. In general, recovery after massive pulmonary embolism is dynamic; ECMO discontinuation or weaning should be guided by an integrated assessment of serial echocardiographic findings, invasive hemodynamics when available, and overall clinical stability rather than any single parameter (30, 36).

The most recent AHA statement on Acute PE states that “*the role of advanced intervention (e.g., mechanical thrombectomy, catheter-directed thrombolysis) for patients on VA-ECMO for acute PE is not known”.* There are no dedicated studies on the current role of percutaneous thrombectomy in combination with short-term VA-ECMO in the treatment of hemodynamically unstable PE. The recent study by Stadlbauer et al. showed in a large and unselected cohort of high-risk PE that advanced recanalization strategies, including systemic thrombolysis, CDT, and surgical embolectomy, resulted in better outcomes (in-hospital mortality of 48%, 43%, and 34%, respectively) compared to an ECMO stand-alone strategy (57%). The CATAPULTE Study Insights (International Cohort) was a large-scale retrospective study of data from international evaluations of early mechanical reperfusion strategies (Percutaneous or surgical thrombectomy) in patients stabilized on VA-ECMO for high-risk PE. These recent data reinforce that upfront stabilization, followed by immediate mechanical or surgical revascularization, significantly decreases early in-hospital mortality.

## Conclusion

Interventional treatment for hemodynamically unstable PE is now common due to the fact that unstable PE carries a significant 30-day mortality. PE is characterized by acute RV failure, hypotension, and hypoxia, leading to cardiovascular collapse and cardiac arrest. The evolving paradigm positions VA-ECMO combined with percutaneous pulmonary mechanical thrombectomy as the preferred combination for the high-risk, hemodynamically unstable PE patients (acute right ventricular failure, refractory shock, and cardiac arrest), while percutaneous pulmonary mechanical thrombectomy alone with standby VA-ECMO suffices for most intermediate, higher-risk PE patients.
